# Severe Dysphagia With Eosinophilic Esophagitis Pattern of Injury Related to Pembrolizumab Therapy

**DOI:** 10.14309/crj.0000000000001252

**Published:** 2024-01-25

**Authors:** James S. Barnett, Kevin K. Yu, Xavier Rivera Rivera, Asmeen Bhatt

**Affiliations:** 1 Department of Internal Medicine, University of Texas Health Sciences Center at Houston, Houston, TX; 2 Center for Interventional Gastroenterology at UTHealth (iGUT), Section of Endoluminal Surgery and Interventional Gastroenterology, Division of Elective General Surgery, Department of Surgery, University of Texas Health Sciences Center at Houston, Houston, TX

**Keywords:** eosinophilic esophagitis, immune checkpoint inhibitor, pembrolizumab, dysphagia, immune related adverse event, PD-1 inhibitor

## Abstract

While immune checkpoint inhibitor (ICI) therapies are effective treatments for many cancers, ICI therapies are associated with immune-related adverse events. We present a 67-year-old man with non–small cell lung carcinoma, who developed severe dysphagia with biopsies from an esophagogastroduodenoscopy showing histopathology consistent with eosinophilic esophagitis while on ICI maintenance therapy with pembrolizumab. The patient's symptoms worsened despite standard therapy. However, he had complete resolution of dysphagia symptoms once pembrolizumab was discontinued. While immune-related adverse events affecting the gastrointestinal system are increasingly recognized, ICI-associated eosinophilic esophagitis is a rare entity.

## INTRODUCTION

Immune checkpoint inhibitors (ICI) are effective therapies for many cancer types, including non–small cell lung carcinoma (NSCLC). While efficacious, ICIs can be associated with immune-related adverse events (irAEs).^1^ We present a 67-year-old man with NSCLC who developed severe dysphagia with an eosinophilic esophagitis (EoE) pattern of injury on histopathology while on pembrolizumab maintenance therapy, with complete resolution of symptoms after discontinuation of ICI therapy.

## CASE REPORT

**Figure 1.**
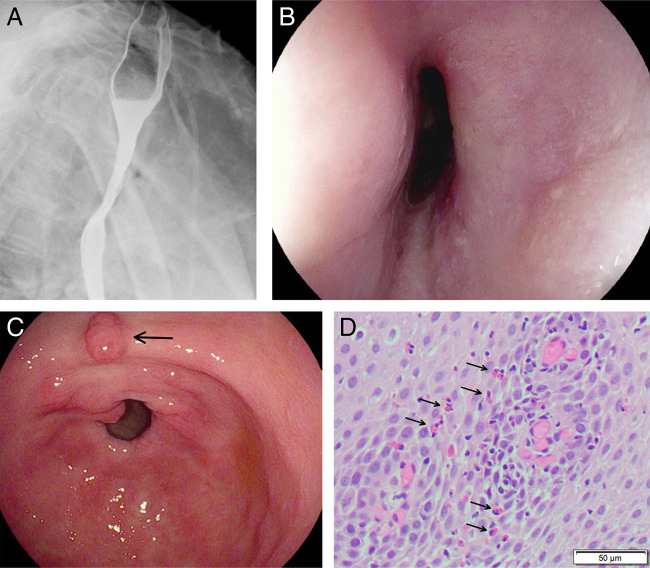
(A) Barium swallow study showing midesophageal narrowing. (B) Endoscopic visualization of the midesophagus showing narrowing of the esophageal lumen. (C) Endoscopic visualization of the gastric antrum and pylorus showing inflammation of the mucosa and a gastric polyp (black arrow). (D) Hematoxylin and eosin stain of esophageal biopsy showing intraepithelial eosinophilia (black arrows) consistent with eosinophilic esophagitis.

A 67-year-old man with medical history of type 2 diabetes mellitus, hyperlipidemia, hypertension, and stable NSCLC on intravenous pembrolizumab therapy 200 mg every 3 weeks for the past 3 years presented to gastrointestinal (GI) clinic with several months of progressively worsening solid and liquid food dysphagia with regurgitation. He denied odynophagia, GI bleeding, weight loss, and no history of allergies or asthma. An esophagogastroduodenoscopy (EGD) completed 2 years before initiating ICI therapy showed normal esophagus and erythema within the gastric body. Biopsies revealed gastric intestinal metaplasia; no esophageal biopsies were collected. Chest computed tomography scan before symptom onset showed stable mediastinal lymphadenopathy with external midesophageal compression. A previous laryngoscopic evaluation by ears, nose, and throat was normal. There were no significant findings on physical examination, and vital signs were normal. Laboratory work was only significant for mild hypokalemia (3.2 mEq/L) and anemia (hemoglobin 11.8 g/dL). A barium swallow study showed midesophageal narrowing in the same area of the known mediastinal lymphadenopathy (Figure 1).

An EGD was then performed and showed midesophageal luminal narrowing, inflammation of the gastric mucosa, and gastric polyp (Figure 1). Esophageal biopsy showed up to 42 intraepithelial eosinophils per high power field (Figure 1), and gastric biopsies revealed gastric intestinal metaplasia without increase in eosinophil infiltrate and negative immunohistochemical stains for *Helicobacter pylori*. Proton pump inhibitor (PPI) therapy twice daily was initiated, but symptoms failed to improve after 10 weeks of treatment. Topical glucocorticoid therapy with budesonide slurry was then initiated. Despite dual therapy for 4 weeks, his dysphagia continued to worsen and the patient self-discontinued his budesonide treatment. In conjunction with the patient's oncology team, a decision was made to discontinue pembrolizumab and start combination therapy with docetaxel and ramucirumab. No dose modifications to pembrolizumab were tried before its discontinuation. At a subsequent clinic visit approximately 1 month after these changes, the patient had complete resolution of dysphagia symptoms and was found to have weight gain of 2.3 kg. He was then transitioned to once daily PPI, without symptom recurrence at follow-up visits. Repeat EGD after symptom resolution was not completed. However, repeat chest CT revealed unchanged mediastinal lymphadenopathy causing external esophageal compression.

## DISCUSSION

This patient's worsening dysphagia in the context of more than 15 intraepithelial eosinophils per high power field without other identifiable etiologies of increased esophageal eosinophils is consistent with EoE. However, his symptoms were refractory to standard therapy for EoE with PPI and topical steroids. The patient's dysphagia could be attributed to external compression from mediastinal lymph nodes; however his lymphadenopathy had been stable for years before presentation. It was only after the discontinuation of pembrolizumab that his symptoms resolved. Although mucosal changes were not visualized during EGD, EoE can cause luminal narrowing and decreased compliance, changes that EGD has poor sensitivity in detecting.^2^ It is possible these changes superimposed on preexisting external compression led to this patient's significant dysphagia. The temporality of symptom onset, treatment failure, and improvement with discontinuation of ICI in the setting of unchanged esophageal compression makes it most likely that this patient's presentation was secondary to ICI induced EoE.

Because the advent of ICIs, irAEs are well documented and continue to be identified.^1^ The most common organs systems affected by ICIs include the skin, GI, and endocrine systems.^3^ The most common irAEs affecting the GI system (GI-irAEs) include diarrhea/colitis (up to 56%),^4^ hepatoxicity (up to 42%),^5^ and pancreatic dysfunction (up to 16%).^6^ While several other GI-irAEs are increasingly recognized, their reported incidence to date is far less, affecting less than 5% of patients.^7,8^ EoE associated with ICIs is a rare entity. In the literature, only 2 other cases of ICI therapy leading to a new diagnosis of EoE have been reported.^9,10^ An additional case was published in which a patient had recurrence of EoE that had previously resolved.^11^ The interval from ICI initiation to onset of GI-irAEs is most often within the first several weeks to months but can occur at any point during treatment.^12^

The general mechanism behind GI-irAEs is poorly understood. It is likely that the drug's ability to ameliorate the immune system inhibits the immune system's ability to be suppressed, leading to various downstream effects.^13^ Histologic findings in acute ICI colitis include neutrophil and/or eosinophil infiltration and increased populations of cluster of differentiation (CD) 4+/CD8+ T cells. The presence and activation of these immune cells likely leads to local inflammation, causing symptoms.^14^ The programmed cell death protein 1 (PD-1) signaling molecule is the target of pembrolizumab and is found on CD4^+^ T-helper type II (Th2) cells. These cells are the primary producer of interleukin-5, which causes eosinophil activation.^15^ Overactivated CD4^+^ Th2 cells leading to interleukin-5 expression and eosinophil activation in patients taking ICIs could be the underlying mechanism driving this patient's disease process.

Treatment of irAEs involves continuation of ICI and symptomatic management for mild symptoms, while management of moderate-to-severe symptoms includes dosing modifications or discontinuation of ICI and initiation of glucocorticoid therapy.^16^ Prophylaxis with biologics and topical steroids for GI-irAEs has been shown to be ineffective in recent studies.^17,18^ Further investigation is needed to identify prevention and management strategies to decrease the incidence and severity of irAEs and prevent the discontinuation of ICI therapy. There is evidence that the incidence of GI-irAEs may depend on the ICI used and at what dose, with PD-1 inhibitors causing irAEs at a lower incidence compared with other ICIs such as cytotoxic T-lymphocyte–associated protein-4 inhibitors or combination immunotherapy with multiple ICIs.^19,20^ As PD-1 inhibitors remain a promising therapy for many malignancies, clinicians should be aware of the GI-irAEs associated with these medications and the appropriate management strategies required to mitigate their effects.

## DISCLOSURES

Author contributions: JS Barnett: lead author. KK Yu: contributing author. XR Rivera: contributing author, contributed pathology images. A. Bhatt: contributing author, faculty supervisor.

Financial disclosure: None to report.

Previous presentation: This case was previously presented at the ACG Annual Scientific Meeting, October 2022, Charlotte, NC.

Informed consent was obtained for this case report.
